# In Silico Screening and Identification of Functional Peptides from Yak Bone Collagen Hydrolysates: Antioxidant and Osteoblastic Activities

**DOI:** 10.3390/ijms26104570

**Published:** 2025-05-10

**Authors:** Yali Wang, Yue Wang, Baishan Fang, Yousi Fu

**Affiliations:** 1 Department of Chemical and Biochemical Engineering, College of Chemistry and Chemical Engineering, Xiamen University, Xiamen 361005, China; wangyal1@msu.edu; 2 Department of Microbiology, Genetics, & Immunology, Michigan State University, East Lansing, MI 48824, USA; 3 MSU-DOE Plant Research Laboratory, US Department of Energy, Michigan State University, East Lansing, MI 48824, USA; 4 Department of Joint Surgery and Sports Medicine, School of Medicine, Xiamen University, Xiamen 361102, China; xmwangyue1992@gmail.com; 5 The Key Lab for Synthetic Biotechnology of Xiamen City, Xiamen University, Xiamen 361005, China; 6 Department of Biochemistry & Molecular Biology, Michigan State University, East Lansing, MI 48824, USA

**Keywords:** collagen peptide, antioxidant peptide, molecular docking, osteogenic activity, MC3T3-E1 cells

## Abstract

Collagen peptides are recognized for their diverse bioactivities; however, efficiently screening potent peptides from hydrolysates remains challenging. This study employed an integrated strategy that combined in silico antioxidant activity prediction and molecular docking to myeloperoxidase (MPO) to screen active peptides derived from yak bone collagen hydrolysates. Focusing on low molecular weight peptides, containing motifs such as GVM, GLP, GPM, and GPQ, we identified nine antioxidant peptides (KC1–KC9). Their activities were validated through in vitro free radical scavenging assays, with peptide KC7 demonstrating superior performance. Furthermore, peptide KC7 promoted proliferation, differentiation, and mineralization in MC3T3-E1 cells by upregulating osteogenic markers such as Runx2 and osteocalcin, modulating the Wnt/β-catenin and PI3K/Akt pathways, and reducing the Bax/Bcl-2 ratio. These results highlight KC7’s dual capacity to mitigate oxidative stress and potentially reduce apoptotic susceptibility, thereby stimulating osteogenesis. This positions peptide KC7 as a promising candidate for bone regeneration and oxidative stress-related disorders. Moreover, this study underscores the effectiveness of integrating computational and experimental approaches for the discovery of multifunctional natural peptides.

## 1. Introduction

Collagen proteins, serve as primary structural components of mammalian extracellular matrices, with particularly high abundance in skin, bones, and connective tissues [1,2]. Collagen peptides, short-chain amino acid derivatives of collagen, have gained significant attention due to their multifunctional properties, including antioxidant, antimicrobial, anti-inflammatory, and immunomodulatory activities [3,4,5]. Driven by the expanding aging population and increasing consumer demand for nutraceuticals and functional foods, the global collagen peptide market has demonstrated exponential growth, reaching an estimated value of USD 2.32 billion in 2023 [6].

Furthermore, collagen peptides have been highlighted for their promising roles in osteoporosis management, capabilities in mitigating oxidative stress and enhancing bone formation [7,8]. An imbalance between reactive oxygen species (ROS) and endogenous antioxidant defenses induces oxidative stress, a key driver in the development of osteoporosis [9]. Excessive levels of ROS trigger a cascade of detrimental effects, including promoting inflammatory responses, accelerating bone resorption, and exacerbating osteoporotic progression [10]. Consequently, interventions targeting oxidative stress have become critical in osteoporosis treatment to mitigate disease progression and improve patient outcomes [11].

Accumulating evidence suggests that the bioactivity of collagen peptides is influenced by their molecular size, with lower molecular weight peptides generally exhibiting greater bioavailability and biological efficacy than their higher molecular weight counterparts [2,12]. The exploration of collagen peptides that promote osteoblast activity has become an increasingly prominent topic in recent research. Wang et al. characterized three antioxidant peptides (GGWFL, ANLGPA, and GWFK) from donkey bone collagen that effectively protected osteoblasts against oxidative damage [13]. Similarly, Chen et al. reported that yak bone collagen-derived peptides (MGF, CF, and MF) exhibited the potential to stimulate osteoblast proliferation, with MGF showing a particularly significant effect, highlighting its potential for osteoporosis treatment [14].

Despite these advances, the rapid screening of highly active peptides from complex collagen hydrolysates remains challenging. In addition to traditional experimental methods, innovative bioinformatics approaches have revolutionized the development of functional peptides [15,16]. Computational tools such as molecular docking, molecular dynamics simulations, and machine learning have been reported in screening and exploring the mechanisms of active peptides [17,18]. For instance, through docking studies, Fan et al. demonstrated that fermented duck liver-derived antioxidant peptides form stable hydrogen bonds with myeloperoxidase (MPO), a key ROS-producing enzyme, exhibiting strong binding affinity that may underlie their oxidative stress reduction capacity [19]. Additionally, Hesamzadeh et al. developed a machine-learning approach for designing antioxidant peptides and identified three novel antioxidant peptides with activities comparable to ascorbic acid [20]. Computational approaches have significantly accelerated antioxidant peptide discovery and increased experimental efficiency, while, more importantly, providing mechanistic insights at atomic resolution. These advancements offer promising solutions for mining bioactive peptides and preventing oxidative stress-related diseases, such as osteoporosis.

In our previous studies, we established an enzymatic hydrolysis method for yak bone collagen complex and identified peptide sequences in the resulting hydrolysates using liquid chromatography–tandem mass spectrometry (HPLC-MS/MS) [21]. However, only a limited number of bioactive peptides have been functionally characterized in vitro and in vivo [21,22,23], and the functions of most identified peptides in the hydrolysates remain largely unexplored. To address this gap, the present study employed a multi-tiered screening strategy combining sequencing screening, antioxidant prediction, molecular docking, and in vitro validation to screen and identify low molecular weight antioxidant peptides. Based on this approach, we successfully explored nine antioxidant peptides (KC1–9). Notably, the peptide GLPGPM (KC7) was found to possess multiple bioactivities, including antioxidant capacity and significant osteogenic effects in MC3T3-E1 cells, by modulating cell proliferation, differentiation, and apoptosis. These findings not only validate our integrated screening approach for antioxidant peptide screening but also highlight KC7 as a multifunctional therapeutic candidate for attenuating oxidative stress and promoting bone regeneration.

## 2. Results and Discussion

### 2.1. Screening Antioxidant Peptides from Yak Bone Collagen Hydrolysates

Our previous study established an enzymatic strategy to generate antioxidant collagen hydrolysates from yak bone collagen using recombinant collagenase derived from *Bacillus cereus*, and characterized more than one hundred peptide sequences in the hydrolysates through HPLC-MS/MS [21]. Among them, a few bioactive peptides were functionally characterized [21,22,23], but we believe that many more functional peptides have not yet been explored. Screening of highly active peptides was challenging. Hereby, we proposed a bioinformatics-driven screening strategy for antioxidant peptides. Firstly, we screened peptides based on bovine bone collagen compositional features. For instance, Gly-Xaa-Yaa sequence units (such as GPA and GPS) are predominantly repeated in bovine bone collagen [24]. What is more, Deng et al. analyzed the composition of 1480 antioxidant peptides and found that proline, leucine, and glycine appeared most frequently [25], and peptides mainly containing these amino acids and motifs have been extensively studied. We therefore focused on identifying low molecular weight collagen peptides, which contain other rare motifs, such as GVM, GLP, GPM, and GPQ in our hydrolysates. Following the antioxidant prediction using both AnOxPP [26] and AnOxPePred [27], nine candidate peptides (KC1–KC9, Table 1) were selected based on their predicted antioxidant potential. Peptide KC7 emerged as the most promising candidate, achieving the highest combined scores in AnOxPePred for scavenger 52.80 × 10^−2^ and chelator score 29.33 × 10^−2^ among the nine peptides.

**Figure 1:**
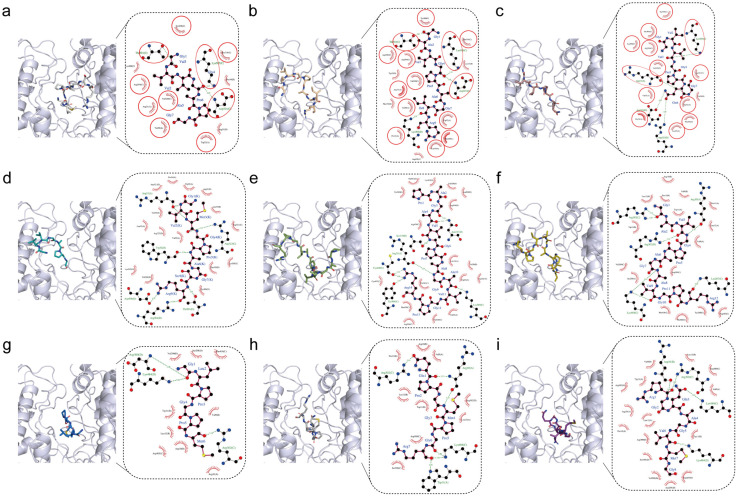
Molecular docking of nine peptides with myeloperoxidase (MPO). (**a**–**i**) Binding conformations and 2D interaction diagrams of MPO-KC1–9 complexes. The Green dashed line in the 2D diagrams (generated by LigPlot+) represented a hydrogen bond. Red eyelash-shaped arcs indicated non-ligand residues participating in hydrophobic interactions.

To further characterize the antioxidant mechanism of candidate peptides, we performed molecular docking against myeloperoxidase (MPO), a neutrophil-derived enzyme that catalyzes reactive oxygen species (ROS) production [28]. While essential for pathogen defense, MPO overactivation can drive oxidative stress and inflammation, making its pharmacological inhibition a strategic target for antioxidants [29]. Studies have shown that antioxidant peptides, with the ability to inhibit MPO, could effectively defend against oxidative stress damage [30]. In terms of binding affinity, all nine peptides bound to MPO with binding affinities below −6 kcal/mol (Table 1), whereas binding affinities below −5 kcal/mol indicate solid binding [31]. Peptide KC5 (PAGAVGPAGAVGPR) exhibited the strongest binding affinity (−8.6 kcal/mol) among the nine peptides, but its binding affinity per residue was the lowest −0.61 kcal/mol/residue (Table 1). Conversely, peptide KC7 demonstrated optimal binding efficiency with the highest per-residue contribution (−1.10 kcal/mol/residue), despite its moderate binding affinity (−6.6 kcal/mol), suggesting superior residue-level complementarity to MPO’s active site (Table 1). Peptides KC1, KC2, and KC3 presented similar GXXGP sequences that were composed of GVVGA, GAVGP, and GVVGP (Table 1), and molecular docking results indicated that they exhibited adjacent binding poses, with their similar N terminus bound to the same surface of MPO (Figure 1a–c). Comparing their interaction diagrams, most residues involved in interacting with peptides KC1–3 via hydrogen bonds or hydrophobic interactions were identical, and Lys505, Thr501, Tyr309, Val320, and Asp321 in chain C and Arg31 in chain A of MPO surround the upper part of peptides KC1–3 (Figure 1a–c, marked by red circles). The peptide KC4 interacted with MPO primarily through hydrogen bonding, including Arg323 (chain C) and Asp508 (chain D) (Figure 1d). For the two relatively longest peptides, KC5 and KC6, there were two more amino acids (P and A) at the N-terminus of peptide KC5. However, their binding conformations were neither close nor similar (Figure 1e,f). The head and tail of peptide KC5 oppositely interacted on the cleavage boundary of MPO, and the middle part was embedded in the cleavage. Meanwhile, Gly1 of peptide KC6 was buried deep in the cleavage forming two hydrogen bonds with Arg302 (chain C) of MPO (Figure 1f). Peptide KC7 with MPO revealed strong interactions through hydrogen bonding, particularly with hydrogen bonds like Gly1 and Asp300/Lys484 (chain D), as well as Met6 and Arg302, suggesting contributing to binding stability (Figure 1g). Additionally, hydrophobic interactions involving Leu2, Pro3, and Pro5, along with electrostatic interactions with Asp300 and Arg10, further enhance the peptide’s affinity for MPO (Figure 1g). Hydrogen-bonding interactions of peptides KC8 and KC9 with MPO were present between Gln and Arg302 (chain C), as well as Trp11 (chain A) (Figure 1h,i).

Through our integrated strategy, we screened nine antioxidant peptides from the hydrolysates and molecular docking revealed their diverse binding modes to MPO. Hence, we synthesized all of them to further determine their antioxidant activities in vitro.

### 2.2. In Vitro Antioxidant Activity Assays

**Figure 2:**
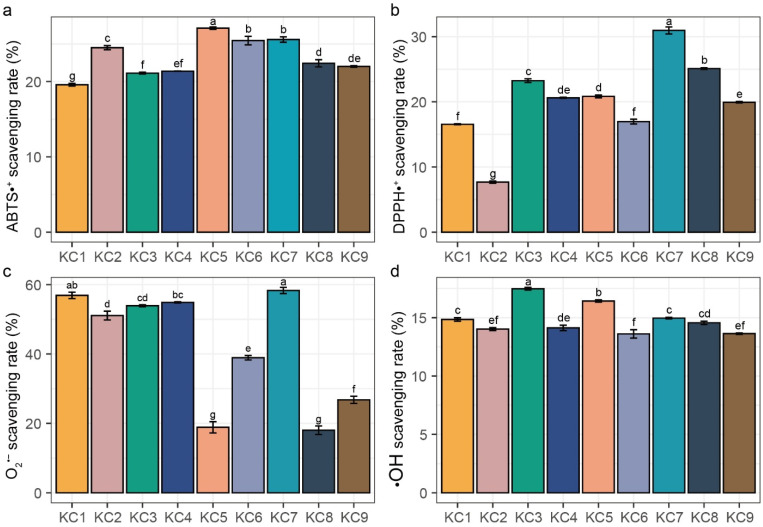
The antioxidant activities of the nine synthesized peptides in vitro. (**a**) ABTS radical scavenging activity, (**b**) DPPH radical scavenging activity. (**c**) Superoxide anion radical scavenging activity. (**d**) Hydroxyl radical scavenging activity. Data were represented as mean ± SD (*n* = 3) and analyzed using one-way ANOVA with Tukey HSD (*p* < 0.05). Groups with different letters indicated significant differences between them.

Given that antioxidant activity arises from multiple mechanisms, and no single assay can comprehensively capture total antioxidant capacity, a comprehensive evaluation requires multiple complementary methods. Current in vitro antioxidant assays are broadly classified into electron transfer (ET; ABTS, DPPH, and superoxide anion scavenging assays) and hydrogen atom transfer (HAT; hydroxyl radical scavenging assay) mechanisms [32]. Our systematic assessment of the nine synthetic peptides revealed significant in vitro antioxidant activities across all the assays (Figure 2). In the ABTS radical scavenging assay (Figure 2a), peptide KC5 demonstrated the strongest activity (27.11 ± 0.16%, *p* < 0.05), followed by peptides KC6–7 (25.45 ± 0.57% and 25.58 ± 0.36%), while peptide KC1 had the weakest activity at 19.59 ± 0.18%. The DPPH radical scavenging assay revealed that peptide KC7 exhibited the highest DPPH radical scavenging activity of 30.96 ± 0.54%, which was significantly higher than the other peptides (Figure 2b, *p* < 0.05). In contrast, peptide KC2 showed the lowest DPPH scavenging ability of 7.68 ± 0.17% (Figure 2b, *p* < 0.05). Regarding the superoxide anion scavenging rate (Figure 2c), peptide KC7 again showed the highest activity of 58.33 ± 0.89%, while peptide KC8 displayed the lowest activity of 18.01 ± 0.89%. The hydroxyl radical scavenging rate ranged from 13.63% ± 0.06% to 17.48 ± 0.12%, with the highest rate occurring at peptide KC3 compared to the other peptides (Figure 2d, *p* < 0.05). The deviation of capacities between individual peptides in the DPPH and superoxide anion scavenging assays were more significant than those in the ABTS and hydroxyl radical scavenging assays, suggesting distinct structure–activity relationships for different antioxidant mechanisms.

After the antioxidant prediction and molecular docking to MPO, selected KC1–9 have been evaluated as antioxidant peptides by in vitro antioxidant activity assays. Peptide KC7 particularly consistently performed prominently across assays. We have proved that KC7 can improve the lifespan and oxidative stress resistance in *Caenorhabditis elegans* [22]. In addition, we also found that the antioxidant collagen peptide GASGPMGPR exhibited osteogenic activities on pre-osteoblasts [23]. This indicated that peptide KC7 had great potential as an effective multi-functional peptide. Hence, we further investigated its roles in osteogenic activity in MC3T3-E1 cells.

### 2.3. Osteogenic Effects of Peptide KC7 on MC3T3-E1 Cells

#### 2.3.1. Assessment of Proliferation, ALP Activity, and Mineralization Capacity

**Figure 3:**
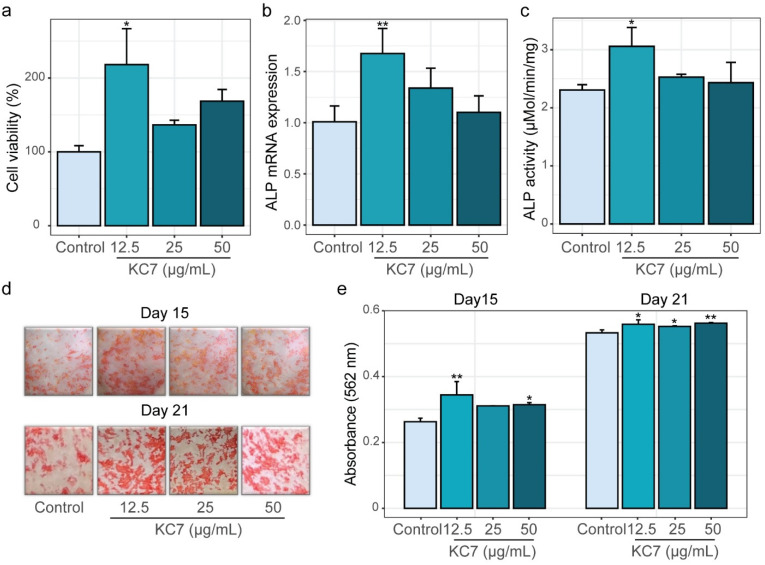
Effects of peptide KC7 on MC3T3-E1 cell viability, differentiation, and mineralization. (**a**) Cell viability assessed by MTT assays. (**b**) ALP mRNA expression levels and (**c**) ALP enzymatic activity. (**d**) Representative images of mineralized node stained by Alizarin Red S on days 15 and 21. (**e**) Quantification of mineralized deposits (absorbance at 562 nm) after 15- and 21-day treatments. The data were represented as means ± SD (*n* = 3) and analyzed by one-way ANOVA with Dunnett’s test, * *p* < 0.05 and ** *p* < 0.01 indicated a significant difference compared to the control group.

Bone metabolism is a dynamic process involving key stages such as proliferation, differentiation, mineralization, and apoptosis, which collectively regulate bone formation and resorption [33]. To systematically evaluate the osteogenic potential of peptide KC7, we first assessed its effects on the proliferation of MC3T3-E1 cells using the MTT assay across three concentrations (12.5, 25, and 50 μg/mL). The results showed that peptide KC7 enhanced cell proliferation, with the best effect at 12.5 μg/mL (Figure 3a, *p* < 0.05). During osteoblast differentiation, the expression of alkaline phosphatase (ALP) serves as a critical early marker of this process [34]. We next investigated peptide KC7’s impact on this process via measuring mRNA expression and enzymatic activity of ALP. At 12.5 μg/mL, peptide KC7 markedly elevated ALP mRNA expression by 1.66-fold (*p* < 0.05; Figure 3b) and the enzymatic activity by 1.33-fold (*p* < 0.05; Figure 3c) compared to the untreated control.

Mineralization, the hallmark of osteoblast maturation [35], was measured on day 15 and day 21 using Alizarin Red S staining. Representative images of stained mineral nodules and the corresponding quantification of dissolved nodules (measured at 562 nm) were presented in Figure 3d,e. Notably, peptide KC7 treatment led to an increased formation of red-stained mineral nodules, indicative of elevated calcium deposition compared to the control (Figure 3d). Quantitative analysis revealed that on the 15th day, 12.5 μg/mL peptide KC7 treatment raised calcium nodule content by 30.97% compared to controls (Figure 3e, *p* < 0.001). This mineralization-promoting effect persisted through the 21st day, with KC7-treated cells maintaining significantly higher calcium deposition levels than untreated controls (Figure 3e, *p* < 0.05). Collectively, these findings demonstrated that peptide KC7 exhibited a remarkable ability to promote osteoblast proliferation, ALP expression, and mineralization in MC3T3-E1 cells.

#### 2.3.2. Alterations in Osteogenesis-Related Gene Expression Induced by Peptide KC7

**Figure 4:**
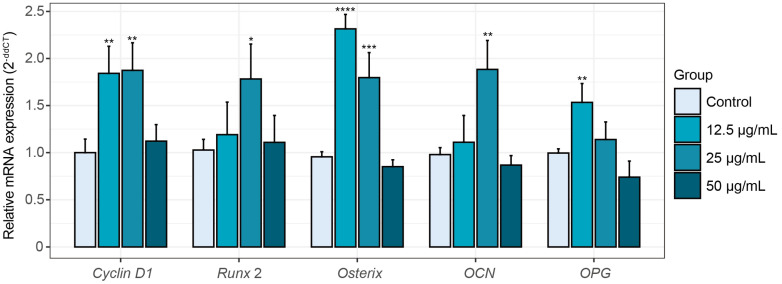
Effects of peptide KC7 on osteogenic gene expression in MC3T3-E1 cells. mRNA expression levels of *cyclin* D1, *runx2*, *osterix*, *OCN*, and *OPG*. Data represented as means ± SD (*n* = 3) and were analyzed by one-way ANOVA with Dunnett’s test, and * *p* < 0.05, ** *p* < 0.01, *** *p* < 0.001, and **** *p* < 0.0001 indicated significant differences compared to the control group.

Osteoblast growth and maturation are intricately regulated by gene expression programs. To further elucidate the osteogenic effects of peptide KC7, we analyzed mRNA expression of key osteogenesis-related genes in MC3T3-E1 cells. Peptide KC7 at 12.5 and 25 μg/mL significantly increased osteogenic marker expression, whereas the 50 μg/mL concentration had no such effect (Figure 4). The proliferation marker cyclin D1, essential for cell cycle progression during osteogenesis, plays a pivotal role in bone formation and osteogenesis [36]. Under 12.5 and 25 μg/mL KC7 treatment, the expression of cyclin D1 was significantly increased to 1.84- and 1.87-fold of the control, respectively (Figure 4, *p* < 0.05). Transcriptional regulators involved in osteoblast differentiation exhibited distinct activation profiles in response to KC7 treatment. Runx2, the master transcription factor for osteogenesis [37], was significantly upregulated by 1.78-fold at 25 μg/mL KC7 (*p* < 0.05). In contrast, osterix (also known as Sp7), which is essential for pre-osteoblast maturation and is regulated downstream of Runx2 [38], was markedly upregulated by 2.42- and 1.80-fold relative to the control at 12.5 and 25 μg/mL KC7, respectively (*p* < 0.05). In addition, functional maturation markers followed this trend, with osteocalcin (OCN, a late-stage differentiation marker) rising 1.88-fold of the control at 25 μg/mL KC7 treatment (*p* < 0.05), indicating enhanced terminal osteoblast differentiation [37]. Peptide KC7 also modulated bone remodeling dynamics via osteoprotegerin (OPG) induction (1.54-fold of the control at 12.5 μg/mL KC7 treatment, *p* < 0.05), which acts as a decoy receptor for nuclear factor kappa-B ligand (RANKL) to prevent osteoclast differentiation and subsequent bone resorption [38]. Together, these results indicated that peptide KC7 functions as a potent osteoinductive agent within the 12.5–25 μg/mL range, promoting the expression of genes associated with proliferation, osteogenic differentiation, and bone resorption inhibition, and further studies will be conducted to elucidate these concentration-dependent mechanisms.

#### 2.3.3. Changes in Osteogenesis-Related Protein Expression Mediated by Peptide KC7

**Figure 5:**
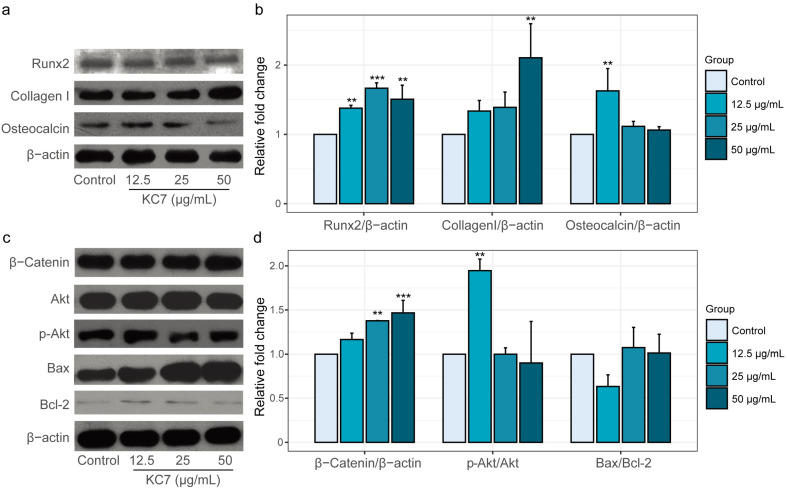
Peptide KC7 modulates osteogenesis-related protein expression in MC3T3-E1 cells. (**a**) Representative Western blot of RUNX2, collagen I, and osteocalcin. (**b**) Quantitative analysis of protein levels shown in RUNX2, collagen I, and osteocalcin. (**c**) Representative Western blot of β-catenin, Akt, phosphorylated Akt (p-Akt), Bax, and Bcl-2. (**d**) Quantitative analysis of β-catenin expression, p-Akt/Akt ratio, and Bax/Bcl-2 ratio. Data represented mean ± SD. ** *p* < 0.01 and *** *p* < 0.001 indicated a significant difference versus the control group.

To corroborate transcriptional regulation with protein expression, we performed a Western blot analysis of key osteogenic factors in KC7-treated MC3T3-E1 cells. Consistent with RT-qPCR results, the highest level of Runx2 expression was observed at a concentration of 25 μg/mL KC7, reaching 1.67-fold of the control group (Figure 5a, *p* < 0.05). Similarly, OCN showed significant protein elevation (1.63-fold of the control at 12.5 μg/mL KC7 treatment, *p* < 0.05), aligning with its mRNA upregulation and confirming KC7’s ability to promote terminal osteoblast differentiation. Peptide KC7 also enhanced the expression of Collagen I, a structural component critical for bone matrix integrity [39]. Intriguingly, Collagen I protein expression reached maximal induction at 50 μg/mL KC7 treatment (2.11-fold of the control, *p* < 0.05). This divergence suggests that the higher KC7 concentration (50 μg/mL) may indicate a shift in KC7’s mechanism of action toward supporting late-stage osteogenic processes, such as matrix maturation and mineralization.

Building upon the observed osteogenic protein induction, we further investigated peptide KC7’s regulatory effects on canonical Wnt signaling and survival pathways. β-Catenin, the central mediator of Wnt signaling governing osteoblast proliferation and differentiation [40], exhibited dose-dependent accumulation with maximal expression at 50 μg/mL KC7 (1.47-fold of the control, Figure 5b, *p* < 0.05). Concurrently, KC7 demonstrated regulation of Akt-mediated survival signaling. While total Akt levels remained stable, the phosphorylated Akt (p-Akt), a critical determinant of osteoblast survival and anabolic activity [41], showed selective activation at 12.5 μg/mL KC7 (p-Akt/Akt ratio: 1.95-fold of the control, Figure 5b, *p* < 0.05). This low-dose enhancement of PI3K-Akt signaling correlated with reduced apoptotic susceptibility [42], as evidenced by a decreased Bax/Bcl-2 ratio (0.63-fold of the control) at 12.5 μg/mL, though statistical significance was not achieved. Bax and Bcl-2 are both members of the BCL2 family, play anti- and pro-apoptotic regulators, respectively, and a decreased Bax/Bcl-2 ratio generally favors anti-apoptotic effects [43]. Our findings indicate that KC7 exhibits concentration-dependent effects on the PI3K/Akt signaling pathway and the concurrent reduction in the Bax/Bcl-2. Several studies have shown that excessive ROS can impair the PI3K/Akt signaling cascade and promote mitochondrial dysfunction and apoptosis [44,45]. Conversely, antioxidant compounds that reduce ROS accumulation have been found to restore p-Akt levels and activate cell survival [46]. Given peptide KC7’s established antioxidant activity, we inferred that lower concentration KC7 activates the phosphorylation of Akt, which helps preserve mitochondrial membrane integrity and ultimately suppresses apoptosis in MC3T3-E1 cells. What is interesting, along with the increased KC7 concentration, the activation effect on Akt rapidly decreased and the Bax/Bcl-2 protein expression was upregulated. We guess even higher KC7 concentration may accelerate cell death, which may be useful in cancer cell disruption. To translate our findings, our future research will thoroughly explore the detailed mechanisms through which peptide KC7 prevents osteoporosis, its therapeutic potential in diseases associated with the ROS/Akt/Bcl-2 signaling axis, and the stability, bioavailability, and optimal delivery of peptide KC7 to determine clinically relevant dosages for bone regeneration.

## 3. Materials and Methods

### 3.1. Molecular Docking Analysis

The 3D structures of the receptor myeloperoxidase (MPO, 1DNU) were obtained from the Protein Data Bank (PDB). These receptor and peptide ligand peptides KC1–9 were prepared by AutoDockTools (version 1.5.7) prior to docking. The original binding sites in the crystal structure were selected as docking sites. Molecular docking simulations were performed with AutoDock Vina (version 1.5.7). During docking, the peptide ligand KC1–9 molecule was designated as flexible with 25 active torsions, while the receptor was set as rigid. Each docking was conducted at least 10 times to ensure statistical robustness. The 2D interactions of the receptor–ligand complex were generated by LigPlot+ (version 2.2.5). The molecular structures were visualized by PyMOL (version 3.7).

### 3.2. In Vitro Antioxidant Activity Assay

Synthetic peptides (>98% purity) were obtained from GenScript (Nanjing, China), and prepared as a 5 mg/mL aqueous stock solution for in vitro antioxidant activity assay.

ABTS^+^ radical scavenging activity was analyzed using the protocol from Song et al. [21]: the 7 mM ABTS (2,2’-azino-bis (3-ethylbenzothiazoline-6-sulfonic acid), Sigma-Aldrich, St. Louis, MA, USA) ethanol solution was mixed with an equal volume of 7.35 mM potassium persulfate, incubated in darkness at 25 °C for 16 h to generate the ABTS^+^ stock solution, which was then diluted to an absorbance of 0.70 ± 0.02 at 734 nm. For testing, 200 μL peptide solution was reacted with 2 mL diluted ABTS^+^ working solution for 20 min in darkness, with distilled water serving as the negative control.

DPPH radical scavenging activity was evaluated according to the method of Brand-Williams et al. [47]: 50 μL peptide solution was combined with 3 mL of 50 μg/mL DPPH (2,2-Diphenyl-1-picrylhydrazyl, Sigma-Aldrich, MA, USA) in methanol, incubated at 25 °C for 60 min in darkness, and absorbance measured at 517 nm using distilled water as the blank.

Hydroxyl radical scavenging activity was assessed via the method of Zhang et al. [48] with a slight modification: the reaction mixture containing 200 μL peptide solution, 70 μL 6 mM H_2_O_2_, 100 μL of 1.5 mM FeSO_4_, and 30 μL of 20 mM sodium salicylate was incubated at 37 °C for 90 min, with absorbance measured at 562 nm. Distilled water was set as a control.

Superoxide anion radical scavenging activity was determined using the pyrogallol autoxidation method [49]: 100 μL peptide solution was pre-incubated with 450 μL Tris-HCl buffer (pH 8.2) for 20 min at 30 °C, followed by the addition of 40 μL 1.26 g/L pyrogallol (Sigma-Aldrich, MA, USA). After 5 min, the reaction was terminated with 100 μL 26.07% HCl, and absorbance was measured at 325 nm. Distilled water was set as a control.

All assays were performed in triplicate using a microplate reader (TECAN, Männedorf, Switzerland). Radical scavenging rates were calculated as Formula (1):

(1) $$Scavenging\;activity\left(\%\right)=\frac{1-\left(Abs.\;of\;sample-Abs.\;of\;blank\right)}{Abs.\;of\;control}\times 100\%$$

### 3.3. MC3T3-E1 Cell Culture

The murine pre-osteoblast MC3T3-E1 cell (ATCC, CRL-2593, Manassas, VA, USA) was cultured in α-MEM basal medium (HyClone, Marlborough, MA, USA) supplemented with 10% fetal bovine serum (FBS, Gibco, Carlsbad, CA, USA) and 1% penicillin–streptomycin (Solarbio, Beijing, China), under standard culture conditions (37 °C, 5% CO_2_, 95% humidity). Cells were routinely subcultured at 80–90% confluence using 0.25% trypsin-EDTA (Solarbio, Beijing, China).

### 3.4. Cell Viability Assay

Cell viability was assessed by the MTT assay kit (Solarbio, Beijing, China) following the manufacturer’s protocol. Briefly, cells were seeded in 96-well plates at 1 × 10^4^ cells/well and allowed to adhere for 24 h. Then, peptide KC7 was administered at concentrations of 12.5, 25, and 50 μg/mL for 24 h. After treatment, 10 μL MTT reagent (5 mg/mL) was added to each well and incubated for 4 h. The supernatant was carefully discarded, and 110 μL formazan solvent was added to each well under gentle shaking for 10 min. Absorbance was measured at 490 nm using a microplate reader (TECAN, Männedorf, Switzerland), with the blank control containing medium-MTT-formazan mixture solution. The cell viability was then calculated using the following Formula (2):

(2) $$Cell\;viability\left(\%\right)=\frac{Abs.\;of\;sample-Abs.\;of\;blank}{Abs.\;of\;control-Abs.\;of\;blank}\times 100\%$$

### 3.5. Alkaline Phosphatase (ALP) Assay

The activity of ALP was determined using an ALP assay kit (Beyotime, Shanghai, China) according to the instruction protocol. Briefly, the cells were seeded in 6-well culture plates at 1 × 10^5^ cells/well and then treated with KC7 (12.5, 25 and 50 μg/mL) for 7 days, with medium refreshed every 48 h. Post-treatment, cells were harvested and washed twice with PBS. Subsequently, cells were lysed with RIPA lysis buffer (Beyotime, Shanghai, China) and centrifuged at 4 °C at 12,000 × *g* for 5 min, and supernatants were collected for analysis. The protein concentration of the supernatant was measured using a BCA assay kit (Solarbio, Beijing, China). The ALP activity of the supernatant was determined by incubating it with p-nitrophenol (10 mM) for 10 min at 37 °C, then stopping the reaction. The absorbance of the reaction solution was read at 405 nm using the plate reader (TECAN, Männedorf, Switzerland). The standard curve of p-nitrophenol was plotted, and net ALP activity was calculated.

### 3.6. Mineralization Assay

The degree of mineralization was determined by the Alizarin red S staining kit (Solarbio, Beijing, China). The medium used for the mineralization study consisted of 50 mg/mL ascorbic acid (Beyotime, Shanghai, China), 10 nM dexamethasone (Beyotime, Shanghai, China), and 2 mM β-sodium glycerophosphate (Beyotime, Shanghai, China) in α-MEM (10% FBS, 1% penicillin–streptomycin) medium. The cells were incubated in 24-well plates and treated with peptide KC7 (12.5, 25, 50 μg/mL) for 21 days, with medium replacement every 3 days. Following the kit’s instructions, the medium was removed, and the cells were washed with PBS. Subsequently, the cells were fixed in 4% paraformaldehyde for 20 min and then washed three times with PBS. Mineralized nodules were then stained with Alizarin S red for 30 min at 25 °C, followed by washes with sufficient Milli-Q water (Millipore, Burlington, MA, USA). After dissolving the stain in the plate with 10% cetylpyridinium chloride (*w*/*v*), the absorbance was observed at 562 nm by the plate reader (TECAN, Männedorf, Switzerland).

### 3.7. RNA Isolation and Real-Time Quantitative PCR

The cells were seeded in 6-well plates in α-MEM with 10% FBS and 1% penicillin–streptomycin for 24 h, followed by peptide KC7 treatment (12.5, 25 and 50 μg/mL) for 7 days. The total RNA was isolated using the total RNA Isolation Kit (Vazyme, Nanjing, China). Complementary DNA (cDNA) was synthesized by HiScript III All-in-one RT SuperMix Perfect for qPCR (Vazyme, Nanjing, China) according to the manufacturer’s protocol. The samples for RT-qPCR were prepared using the SYBR qPCR Master Mix (Vazyme, Nanjing, China). Primer sequences for target genes are listed in Table S1. The quantified results were calculated using the 2^−ΔΔCt^ method.

### 3.8. Western Blotting Assay

MC3T3-E1 cells were treated with peptide KC7 at concentrations of 12.5, 25, and 50 μg/mL for 7 days as previously described. Post-treatment, cells were harvested, washed with ice-cold PBS, and lysed in RIPA lysis buffer (Beyotime, Shanghai, China) supplemented with 1× protease and phosphatase inhibitor cocktail (Beyotime, Shanghai, China). Total protein concentrations were quantified using a BCA assay (Solarbio, Beijing, China), and 40 μg of protein lysate per sample was resolved on 15% SDS-PAGE gels. Separated proteins were electrophoretically transferred to polyvinylidene fluoride (PVDF) membranes, followed by blocking with 5% nonfat dry milk and 1% BSA in Tris-buffered saline containing 0.1% Tween-20 (TBST) for 1 h at room temperature. Membranes were incubated overnight at 4 °C with the following primary antibodies (all from Abcam, Oregon, USA): anti-Runx2 (ab236639), anti-Collagen I (ab255809), anti-Osteocalcin (ab133612), anti-Akt1 + Akt2 + Akt3 (phospho S472 + S473 + S474, ab192623), anti-Akt1 + Akt2 + Akt3 (ab200195), anti-β-Catenin (ab6301), anti-Bax (ab182734), and anti-Bcl-2 (ab16904). After three TBST washes, membranes were incubated with HRP-conjugated secondary antibodies (anti-β-actin, Sangon Biotech, Shanghai, China) for 50 min at room temperature. Protein bands were visualized by ChemiDoc Touch (BIO-RAD, Hercules, CA, USA). The results of band assays were expressed as fold change relative to the corresponding untreated control.

### 3.9. Statistical Analysis

All data results were presented as mean ± standard deviation. All tests were repeated at three replications. One-way analysis of variation (ANOVA) followed by Dunnett’s test was utilized in data analysis by R (version 4.3.3), with *p* < 0.05 indicated as significant.

## 4. Conclusions

This study successfully identified nine antioxidant peptides from yak bone collagen hydrolysates using an integrated strategy that combined bioinformatic antioxidant screening, in silico molecular docking to myeloperoxidase (MPO), and in vitro validation on free radical scavenger. Beyond the antioxidative properties, peptide KC7 (GLPGPM) significantly enhanced osteogenic activity in MC3T3-E1 cells by promoting proliferation, differentiation, and mineralization. Mechanistically, KC7 upregulated critical osteogenic markers, including Runx2 and Collagen I, via the activation of the PI3K/Akt/Bcl-2 signaling pathway, thereby fostering bone matrix maturation. These findings position the antioxidant peptide KC7 as a promising candidate for addressing oxidative stress-related disorders and bone regeneration challenges. Furthermore, this work provides an efficient approach for the rapid development of natural functional peptides from collagen hydrolysates.

## Figures and Tables

**Table 1:** Characterization, antioxidant activity prediction, and molecular docking results of nine peptides.

Name	Peptide Sequence	Molecular Weight	Yak Collagen Resource	AnOxPP	AnOxPePred		AutoDock Vina	
				Pre-Score (10^−2^)	Scavenger Score (10^−2^)	Chelator Score (10^−2^)	Affinity (kcal/mol)	Per-Residue Affinity (kcal/mol)
KC1	GVVGAPG	555.30	alpha-2(I)	99.81	43.12	21.58	−6.7	−0.96
KC2	GAVGPAGIR	796.46	alpha-2(I)	99.99	45.10	22.69	−7.2	−0.80
KC3	GVVGPQG	612.32	alpha-2(I)	99.80	45.22	23.80	−6.9	−0.99
KC4	GVMGPAGSR	830.41	alpha-2(I)	99.99	46.74	21.54	−7.0	−0.78
KC5	PAGAVGPAGAVGPR	1217.65	alpha-2(I)	99.99	50.96	19.70	−8.6	−0.61
KC6	GAVGPAGAVGPR	1007.55	alpha-2(I)	99.99	47.57	18.18	−8.1	−0.68
KC7	GLPGPM	570.71	alpha-1(XIII)	99.93	52.80	29.33	−6.6	−1.10
KC8	QPGMPGR	782.82	alpha-1(IV)	99.98	47.04	26.20	−7.2	−1.03
KC9	RGQAGVMG	774.90	alpha-1(I)	99.16	38.12	20.15	−6.5	−0.81

## Data Availability

All data have been included in the manuscript.

## Supplementary Materials

The following supporting information can be downloaded at: https://www.mdpi.com/article/10.3390/ijms26104570/s1.
