# Supplementary material for: In Silico Screening and Identification of Functional Peptides from Yak Bone Collagen Hydrolysates: Antioxidant and Osteoblastic Activities
Source: Int J Mol Sci. 2025 May 10;26(10):4570. doi: 10.3390/ijms26104570 (PMC12111753; doi:10.3390/ijms26104570)
Supplement: Supplementary file 1 [file ijms-26-04570-s001.zip › ijms-3623369-supplementary.pdf]

Table S1. Primers for RT-qPCR.

Gene	Forward (5′ → 3′)	Reverse (5′ → 3′)
ALP	CCAACTCTTTTGTGCCAGAGA	GGCTACATTGGTGTTGAGCTTTT
Akt	ATGAACGACGTAGCCATTGTG	TTGTAGCCAATAAAGGTGCCAT
cyclin D1	AACTACCTGGACCGCTTCCT	CCACTTGAGCTTGTTCACCA
Runx2	CCGATGGGACCGTGGTT	CAGCGTCAACACCATCATTC
Osterix	CTGGAAGGGCTGGGTAAAC	AGGGCAGGTATGGCTTCTTT
Osteoprotegerin	TGAAGACCTACCTGCAACGT	TGCAAACACACACTCATCAC
Osteocalcin	CTCTGTCTCTCTGACCTCACAG	GGAGCTGCTGTGACATCCAT
β-actin	GGCTGTATTCCCCTCCATCG	CCAGTTGGTAACAATGCCATGT
